# Association Is Not Prediction—A Pervasive Issue in the Medical Literature

**DOI:** 10.1111/cea.70040

**Published:** 2025-03-26

**Authors:** Kaitlin Stangroome, Michael R. Perkin

**Affiliations:** ^1^ St. George's University of London London UK; ^2^ Population Health Research Institute St. George's University of London London UK

**Keywords:** allergens and epitopes, education, ethics, prevention, regulatory aspects

**Summary**

- Only 17% of papers in the allergy field claiming prediction reported sensitivity and specificity.
- Clinical practice and patient care can be compromised by erroneous assertions of predictive value.

To the Editor,

It has recently been observed by Varga et al. that the available scientific literature ‘demonstrates a common tendency to claim predictive value from studies aimed at determining associations’ [1]. While research studies often uncover numerous associations, associations themselves do not convey predictive value and ‘confusion between association and prediction harms clinicians, scientists, and ultimately, the patients’ [1]. Varga et al. systematically reviewed published papers in the field of diabetes epidemiology that made claims about prediction in their title and assessed whether they reported findings with proper relevant measures of prediction in their abstract [1]. The purpose of their research was to identify titles where certain biomarkers were evaluated for their predictive ability in relation to diabetes *as an outcome*, or where diabetes would predict *another outcome* (e.g., disease progression, response to treatment or the development of complications) [1]. They found that 61% of papers referring to prediction in their titles did not report metrics of predictive statistics in their abstracts [1]. In a simulation data exercise they demonstrated that biomarkers with large effect sizes and statistically significant *p* values can still offer poor discriminative utility [1]. We undertook a systematic review to see if similar results are seen in the field of allergy epidemiology, acknowledging that disease prediction is a much more established area of research for diabetes than it is for allergy. We used the same categorisation of statistical methodologies as Varga et al. [1] to identify measures of prediction and measures of association.

A PubMed search was conducted on the 25th of May 2023 to identify studies published between February 1981 and May 2023 that met the search criteria. The aim was to find studies which contained prediction in their titles alongside an allergy descriptor. The search retrieved 1003 titles, of which 173 were subsequently excluded, leaving 830 abstracts and titles. Abstracts were then divided according to content. The 830 abstracts were categorised by allergic condition and area of investigation.

Using the criteria specified in the Varga et al. paper, we identified those with prediction metrics and then those with association metrics. Those containing neither measures of prediction nor association were designated as undefined. Overall, only 39% of the studies (323/830) reported prediction metrics in their abstracts. The remaining 61% (507/830) were divided between 38% (317/830) which reported methods of association and 23% (190/830) that did not report a clear methodology (undefined). Only 17% of the studies reported sensitivity and specificity in their abstracts, with 142/830 reporting sensitivity and 139/830 reporting specificity.

The distribution of allergic conditions across our categorisation of statistical methodologies used (prediction metrics, undefined, association metrics) was broadly similar. However, the areas of investigation did differ. A larger proportion of abstracts with prediction metrics focused on modelling and diagnosis, compared with those without prediction metrics. In contrast, a larger proportion of papers utilising association metrics focused on risk factors compared to papers using prediction metrics.

Amongst prediction metrics, the most common metric was ROC AUC (Receiver Operating Characteristics Area Under the Curve) with 165 mentions (Figure 1). There were 26 different prediction metrics used (range 1–165 abstracts). For association metrics, the most common metric was logistic regression with 147 mentions, out of 17 different methods of association used (range 1–147 abstracts) (Figure 1).

**FIGURE 1:**
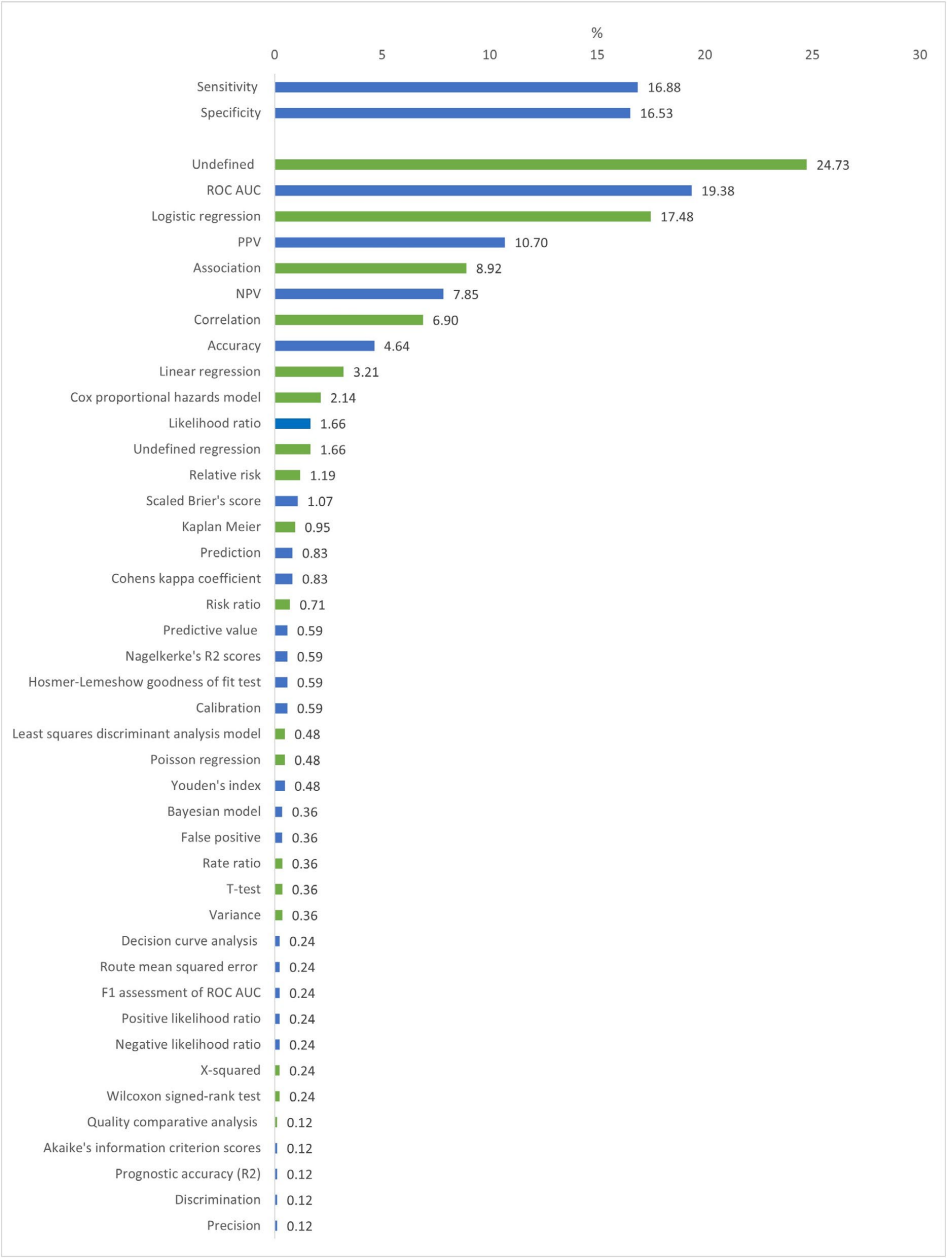
Percentage of reported methods in abstracts analysed (*N* = 830). Blue: Percentage of reported methods in abstracts considered metrics of prediction by Varga et al. (*N* = 323). Green: Percentage of reported methods in abstracts considered not to be metrics of prediction by Varga et al. (*N* = 507).

In conclusion, most studies in the field of allergy that claim to show prediction provide no evidence of prediction metrics within their abstracts. Abstracts were more likely to include predictive metrics where the subject area was modelling or diagnosis. Where risk factors have been identified as associations in, for example, a cohort study, and predictive ability has been inferred, the use of the term prediction has the potential to have been used erroneously.

The strength of our approach was using a comprehensive search strategy previously used across another field of medicine. The principal limitation is that our research was unfunded, and we were not able to undertake a full text analysis or any form of sensitivity analysis by underlying condition or investigation. However, Varga et al. did undertake a full text analysis of 100 papers they had identified and which had not reported metrics of prediction in their abstract and found that only 15 did contain metrics of prediction in the full text [1]. The concern that the distinction between measures of association and prediction is not appreciated has been raised by others [2, 3, 4]. The 39% figure for the allergy papers analysed providing metrics of prediction is identical to the 39% figure observed in the field of diabetes [1]. Additionally, even fewer of the papers reported sensitivity and specificity metrics. This consistency suggests that this is likely to be a pervasive issue across medical disciplines.

Many of these papers promise improvements in patient care and this could lead to inflation of the clinical importance of their findings. Patient care can potentially be compromised by statistically untrained clinicians incorrectly inferring clinical prediction when such evidence is absent. If prediction is being used appropriately then sensitivity and specificity are pre‐requisites in the context of the ability of a measurement to predict an outcome and their presentation facilitates comparison between diagnostic tools [5, 6]. Tighter statistical scrutiny should be advocated to avoid similar problems in the future and EQUATOR reporting guidelines such as TRIPOD (Transparent Reporting of a multivariable prediction model for Individual Prognosis or Diagnosis) should be used where appropriate [7].

## Author Contributions

M.R.P. contributed the original idea. K.S. and M.R.P. contributed to the study design. K.S. conducted the data search, analysed the data, prepared study results, and drafted the manuscript. K.S. and M.R.P. contributed to revising the manuscript and approved the final version.

## Conflicts of Interest

The authors declare no conflicts of interest.

## Data Availability

The search terms used, reasons for exclusion and categorisation of the retrieved abstracts by allergic condition and area of investigation are available in the Figshare Online Repository (10.24376/rd.sgul.28539824).
